# Oswestry Hospital Scandal

**Published:** 1888-02-11

**Authors:** M. Mattocks

**Affiliations:** Oswestry Cottage Hospital


### THE EDITOR'S LETTER-BOX.

### THE OSWESTRY HOSPITAL SCANDAL.

Will you kindly permit me to correct a sensational paragraph respecting the rules of the Oswestry Cottage Hospital, evidently supplied by some highly imaginative correspondent? In the first place, the hospital rules are *unlike* the laws of the Medes and Persians in that they can be, and are, altered whenever the committee sees sufficient reason, and relaxed at once when the special urgency of any case requires it, though it is true that they are neither altered nor relaxed to suit the private whim or convenience of any individual. Next, the child, instead of being brought here to be dressed, which would have been done at once, was brought by its mother, after being dressed at home by a doctor, who told her to take it to the hospital, which she did against her own judgment, as she knew the rule very well. Whether taking a three-year-old child from its mother and keeping it with strangers in a strange place would be likely to "soothe" it, I leave to the judgment of anybody who knows young children. As to the burns, which I dressed myself the next day, they were only such as would be treated in the out-patients' department of any hospital. Neither the non-existent hall-porter nor the non-resident chairman of committee can be held responsible for an occurrence of which they were equally ignorant. The matron, who is held up to reprobation, was at that very time lying dead in the hospital, and the sister who was doing her work had in her own grief and the care of the hospital enough to bear without the addition of a gratuitous worry, which no one possessed of common human instinct would have chosen that time to inflict on her.

M. Mattocks.

Oswestry Cottage Hospital, Feb. 6th, 1888.

[We are glad to receive this quite satisfactory explanation. —Ed.]

